# Stepping Up Medicinal Chemistry Capabilities in Sub-Saharan Africa: Perspectives from Early Career African Drug Discovery Researchers

**DOI:** 10.1021/acs.jmedchem.5c00081

**Published:** 2025-05-12

**Authors:** Godfrey Mayoka, Peter Mubanga Cheuka

**Affiliations:** † Helmholtz Institute for Pharmaceutical Research, Helmholtz Centre for Infection Research, 66123 Saarbrucken, Germany; ‡ School of Pharmacy, Jomo Kenyatta University of Agriculture and Technology, 62000-00200 Nairobi, Kenya; § Department of Pure and Applied Chemistry, School of Natural and Applied Sciences, University of Zambia, 32379 Lusaka, Zambia

## Abstract

Despite Africa’s progress in basic and clinical sciences, gaps persist in its translational capacities, especially medicinal chemistry, a cornerstone of early stage drug discovery. Drawing on experiences as early career researchers and alumni of Africa’s premier holistic drug discovery and development center (H3D), this perspective highlights key successes, challenges, and opportunities in Africa. Major barriers to drug discovery in Africa include limited hit-to-lead and lead optimization programs, insufficient Africa-tailored funding, and educational curricula misaligned with real-world drug discovery. We advocate scaling successful models, integrating drug discovery concepts into educational curricula, and forming strategic partnerships with leading African and international institutions. Africa’s progress in drug discovery requires innovative funding mechanisms and resource-sharing frameworks adapted to its unique challenges. Targeted investments, collaborative networks, and reciprocal knowledge exchange are essential for Africa to transition from a consumer of global medical innovations to a leading contributor, addressing local healthcare needs and advancing global health equity.

## Graphical abstract

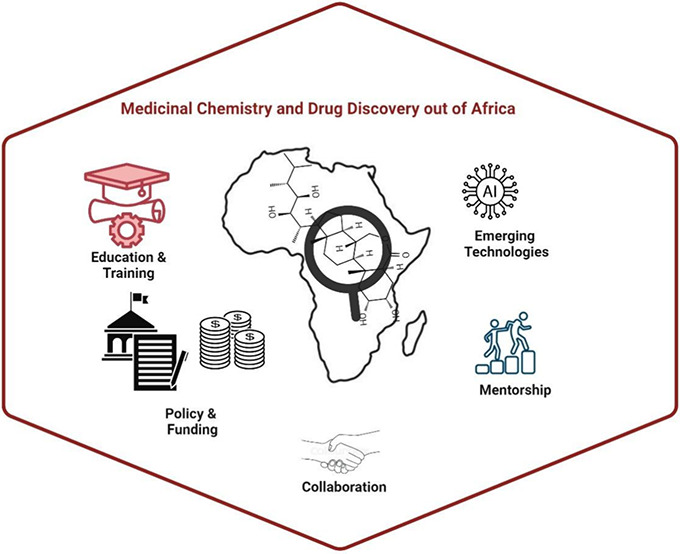

## Significance

- Significance: This perspective underscores Africa’s need to bridge translational gaps in drug discovery, particularly in medicinal chemistry, to advance local and global health solutions.
- Impact: By advocating for research hubs, curriculum integration, and strategic partnerships, the article outlines actionable steps to strengthen Africa’s drug discovery ecosystem.
- Innovation: This article highlights creative and tailored funding mechanisms and resource-sharing frameworks, providing realistic solutions to the pressing funding and infrastructure challenges in Africa.

## 1 Introduction

Africa is home to nearly 20% of the global population but has yet to make a proportionate impact on global research and innovation. Current data reveal that Africa contributes only 2–3% of global scientific output, with investments in research and development (R&D) falling below what is required in addressing the continent’s immense healthcare and socioeconomic challenges.^1^ This shortfall is particularly alarming given Africa’s disproportionate disease burden, which includes over 85% of global cases of neglected tropical diseases, alongside significant rates of HIV/AIDS, tuberculosis, malaria, and a rising prevalence of noncommunicable diseases such as diabetes and cancer. These conditions collectively exacerbate the continent’s healthcare crisis, underscoring the urgent need for more proactive involvement of African researchers in addressing these challenges.^2^

Despite the diversity and scale of healthcare needs, Africa’s contribution to the drug development pipeline remains predominantly limited to late-stage clinical trials, which are equally small. These limited clinical trials of medicines before they are deployed raises critical concerns about the suitability of such medicines for the African population, given the continent’s unique genetic, geographical, and socio-economic contexts.^3,4^ To ensure effectiveness and safety, medicines must be optimized for African patients and tailored to address regional healthcare priorities which demands the active involvement of African scientists at the forefront of drug discovery efforts.^5^

Africa’s current landscape reveals strengths in basic sciences and clinical implementation. However, there is a critical gap in translational science, particularly in medicinal chemistry, the cornerstone of early drug discovery.^6^ Developing robust medicinal chemistry capabilities is essential for bridging this gap and establishing a self-reliant drug discovery ecosystem. Such efforts would enable Africa to address its diverse disease challenges while contributing to global health equity. Moreover, integrating medicinal chemistry into broader R&D frameworks promises to generate cross-therapeutic applications, fostering innovation that supports socioeconomic development and aligns with the United Nations’ Sustainable Development Goals.^7^

Africa is making promising strides in strengthening its early drug discovery capabilities. However, these efforts remain largely concentrated in North Africa, exemplified by Egypt, and South Africa, where several universities have developed a strong medicinal chemistry infrastructure. Among them, the H3D center,^8^ at the University of Cape Town (UCT), has emerged as a flagship institution, integrating academic training in medicinal chemistry with industry-level drug discovery projects. Outside North Africa and South Africa, initiatives such as the West African Centre for Cell Biology of Infectious Pathogens (WACCBIP)^9^ and the Drug Innovation Group in Ghana^10^ are providing additional training opportunities for emerging researchers, contributing to human capital resource development. These pioneering efforts highlight Africa’s potential to build a critical mass of researchers capable of driving sustainable and impactful R&D.

As early career researchers and alumni of H3D, we bring a perspective to the discussion on stepping up medicinal chemistry capabilities on the African continent. Our journey through medicinal chemistry training and drug discovery research has afforded us firsthand insights into the challenges and opportunities in navigating this field. By sharing our experiences, ranging from dealing with challenges associated with resource constrained institutions to leveraging international collaborations, in addition to teaming up between ourselves, we aim to inspire a new generation of African scientists to embrace the challenges associated with mounting and executing drug discovery research in Africa and contribute to solving the continent’s health and, by extension, socioeconomic challenges.

In addition to sharing our early career experiences, we advocate for the deliberate scaling of efforts to replicate and expand the successes achieved at H3D and other aspiring centers across the continent. Building a vibrant medicinal chemistry landscape in Africa must be a purposeful, multisectoral endeavor to empower the continent to tackle its burgeoning healthcare challenges. This requires concerted efforts from policymakers, academic institutions, and global partners to support the growth of African-led initiatives in medicinal chemistry. By fostering these efforts, we can pave the way for a new era of Afrocentric drug discovery, transforming Africa from a long-held position, as a recipient of medical solutions or a late entrant into the drug development space, into a leader in addressing global health challenges.

## 2 Personal Experiences on Navigating the Drug Discovery Terrain in Sub-Saharan Africa

### 2.1 Godfrey Mayoka

Completing my bachelor’s degree in pharmacy at the University of Nairobi marked a pivotal starting point for my career. At that time, traditional career options in Kenya for pharmacy graduates were primarily limited to community, hospital, or industrial quality assurance, quality control, or regulatory affairs roles, which often lacked research and development opportunities. Informed by my analytical observations of older colleagues, the career choices they made and prospects that they had or missed, and driven by a passion for academia and research, I decided to pursue a career that would allow me to contribute to drug discovery. However, the pathway to postgraduate studies in drug discovery was not straightforward due to the lack of drug discovery-related programs at the time.

My first opportunity to engage in preclinical research came during a research assistantship at the African Institute of Biomedical Science and Technology (AiBST) in Zimbabwe.^11^ In this role, I was exposed to practical research in pharmacogenetics, particularly focusing on drug–drug interactions in HIV, TB, and antiparasitic treatments among African populations. This hands-on experience proved invaluable when I later applied for a Commonwealth scholarship to pursue a Master’s in Drug Discovery at University College London (UCL) in the UK. The program at UCL provided foundational training in drug discovery, reinforced by industry visits and lectures by pharmaceutical industry experts.

After returning to my home country, as required of recipients of a Commonwealth scholarship upon completion of their studies, I secured a teaching position at Jomo Kenyatta University of Agriculture and Technology (JKUAT). However, the limited infrastructure for practical drug discovery research locally prompted me to seek further training opportunities for my doctorate elsewhere, leading to my admission at UCT. My time at UCT, under the mentorship of Prof. Kelly Chibale, was pivotal in consolidating my knowledge and practical involvement in drug discovery, marking a critical turning point in my career. The unique environment at H3D, which bridges academic drug discovery with industry-level projects, had a profound impact on shaping my training experience and inspiring my thoughts for the future. I embraced the opportunity to work on antimalarial and antischistosomal drug discovery, based on phenotypic-based hit compounds.^12–14^ My doctoral training was further enriched by conference participation and presentations, sharing my work with other researchers, thereby helping build a network of collaborations that has been invaluable for my professional growth.

Toward the end of my Ph.D., I was acutely aware of the stark contrast in research resources that awaited me when I return to my home institution, a far cry from the enabling research atmosphere at UCT. I knew I had to seek opportunities to sustain my participation and contribution to drug discovery and related pharmaceutical sciences or risk being overwhelmed by teaching responsibilities or, at best, administrative roles. With the support of the Africa Research Excellence Fund (AREF),^15^ I pursued a postdoctoral fellowship at H3D, where I expanded my experience to include target-based drug discovery in antimalarial research and participated in collaborative projects, honing essential project management and networking skills. During this time, I also mentored students and guided them in research publication, an effort that led me to establish an academic and scientific research writing program at my home institution to empower young scientists in scholarly writing.

Overall, my experience at H3D was transformative, exposing me to advanced research environments and the strategic importance of collaborations in advancing the frontiers of African-led drug discovery initiatives. Attending H3D symposia^16^ further underscored the importance of networks and partnerships, which eventually led to my current postdoctoral position in antibiotic drug discovery at the Helmholtz Institute for Pharmaceutical Research Saarland in Germany. This role has equipped me with additional skills in target-based drug discovery and dynamic combinatorial chemistry, broadening my scope of research methodologies.

Looking forward, I aspire to leverage these experiences to foster research networks and expand funding opportunities for early career researchers in Africa. I have been engaged in supporting African scientists through mentorship and collaborative projects, particularly in underrepresented fields such as drug discovery for endemic African diseases. Together with former colleagues, who were my contemporaries during my Ph.D. training, we have coauthored research articles and reviews and participated in cosupervision of postgraduate students in medicinal chemistry and share a common goal of contributing to a stronger foundation for medicinal chemistry and drug discovery on the continent, promoting local talents and enhancing global partnerships.

### 2.2 Peter Mubanga Cheuka

After completing my undergraduate degree at the University of Zambia (UNZA), I was expected to pursue postgraduate training as a physical chemist. As opportunities in this area were not forthcoming, I reconsidered my options and explored chances in medicinal chemistry and drug discovery research, leading to an offer by Prof. Kelly Chibale, at UCT. Here, I pursued my M.Sc. studies with a focus on medicinal chemistry optimization of small organic molecules for potential application as new antimalarials. Witnessing the progression of projects from discovery phases through preclinical testing to clinical trials at H3D ignited my passion for drug discovery.

After completing my M.Sc. studies in 2014, I was appointed as a full-time lecturer at UNZA, where I soon realized I needed Ph.D. training to acquire relevant technical and soft skills that would spur my career growth. Between 2015 and 2019, I pursued my Ph.D., again under Prof. Chibale. This Ph.D. journey has positively shaped my career progression in profound ways. Having a supervisor with an established track record and maintaining a collaborative relationship poststudentship has been crucial to my career progression. Prof. Chibale has continued supporting my efforts in getting established as a researcher in different ways ranging from coauthoring review manuscripts to providing access for conducting experiments for which I do not have the capacity in my current research environment. In fact, this collaboration was finally formalized in early 2024 through a collaborative agreement signed between UNZA and UCT that allows H3D to host UNZA postgraduate students on research visits. So far, one student from my research group has already visited H3D for 6 weeks, enabling the student to make rapid progress in the synthesis of small antischistosomal and antimalarial organic molecules.

Apart from exposure to the international research community through attendance at conferences and authoring some of my most influential papers yet,^17–21^ the Ph.D. experience provided another career-defining opportunity, being selected to participate in the Next Generation Scientist (NGS) program.^22^ Administered by Novartis AG in conjunction with University of Basel, the program provides three months of training in scientific and clinical research to talented and motivated scientists from emerging countries. In addition to acquiring skills that I continually apply in my teaching and research, I was introduced to WIPO Re:Search, a consortium then sponsored by the World Intellectual Property Organization (WIPO) in collaboration with BIO Ventures for Global Health (BVGH).^23^ Among the goals of this consortium was to promote collaborations between institutions in order to accelerate R&D against neglected tropical diseases. After facilitating the onboarding of my own home institution, UNZA, onto this consortium, I established a number of collaborations with other institutions, among them, the Centre for Discovery and Innovation in Parasitic Diseases (CDIPD) at University of California San Diego (UCSD). This collaboration has been the most successful yet and continues to be pivotal in the progress my research group is making in antischistosomal drug discovery.

The decision to start medicinal chemistry projects that are now flourishing was a difficult one, requiring a lot of determination and self-drive. When I completed my Ph.D. in 2019, I faced the dilemma of either going back to my home country to resume my duties at UNZA or staying in South Africa or migrating to the global north for better opportunities. Against the tide, I chose to give a chance to establish my career in Zambia, motivated by the need to reduce the brain drain that Africa continues to experience. After initial diversion of research interests due to resource and infrastructure limitations, stumbling on some chemicals and reagents in the departmental chemical store turned on the lights that started my medicinal chemistry research at UNZA. We synthesized the first set of compounds using these chemicals and reagents and tested them for *in vitro* antischistosomal activity through my collaborator, Prof. Conor Caffrey of the CDIPD at UCSD. The preliminary data from this work was central in securing grant funding amounting to EUR 30,000 from Merck KGaA, further expanding our medicinal chemistry capacity and contribution to the antischistosomal drug discovery community by reporting the first ever set of compounds made in our laboratories.^24^ Through a recent research visit to AiBST in Harare, Zimbabwe, I carried out *in vitro* drug metabolism and pharmacokinetic (DMPK) studies on some of the front-runner compounds. This visit was supported by grant funding of USD 10,000 from AiBST and the Ministry of Higher and Tertiary Education Innovation Science and Technology Development of Zimbabwe, under the SPARK Africa program, whose focus is to help convert discoveries into clinical solutions for African populations.^25^ Together with gaining full membership to the Grand Challenges African Drug Discovery Accelerator (GC ADDA) consortium, a network of drug discovery researchers, supported by the Gates Foundation,^26^ these opportunities have accelerated progress in my drug discovery projects. This membership allows me to utilize the network’s drug discovery facilities, collaborate with other scientists, receive sponsored invitations to annual meetings; and nominate my students for training, mentorship, and short-term scientific placements. The latest achievement is receiving, alongside 8 other GC ADDA member institutions, a European Union grant funding amounting to close to EUR 1.5 million over a period of three years; of which about EUR 85,000 has been allocated to UNZA. This funding will facilitate the recruitment of two Ph.D. students into my research group in 2025 and support further capacity building for early drug discovery in Africa.

Building on the growing experience of my research group, I intend to continue attracting additional funding to enable me to recruit more senior postgraduate students and retain some as postdoctoral fellows to mentor younger researchers. In establishing and strengthening partnerships with colleagues on the continent, I am confident that we will contribute to the drug discovery footprints in Africa.

## 3 Perspectives on Strengthening Medicinal Chemistry in Africa

### 3.1 Education and Training

#### 3.1.1 Quality and Relevance of Training Programs

There is generally a dearth of graduate programs focused on providing foundational knowledge and skills in medicinal chemistry and drug discovery, at African universities. Closely related programs have significant gaps in their adequacy and relevance with many curricula often relying heavily on basic principles, often disconnected from practical applications in addressing pressing health challenges unique to the continent. The lack of alignment with modern drug discovery processes reflects a broader issue surrounding the limited exposure of many instructors to real-world pharmaceutical R&D. This disconnect restricts students’ ability to contextualize their theoretical knowledge, leaving them underprepared for industry roles and globally competitive and impactful research initiatives.

To bridge this gap, African universities must modernize their curricula, emphasizing experiential learning and case-relevant applications. Practical exercises, case studies, and laboratory simulations should mirror challenges encountered in pharmaceutical development. Partnerships with pharmaceutical companies actively engaged in R&D can provide students with the necessary hands-on experience, equipping graduates with skills that meet contemporary drug discovery demands. Strengthening these collaborations will enable students to seamlessly transition into the workforce, minimizing the need for extensive onboarding and enhancing their competitiveness on the global stage. In addition, practical skills obtained during the training can inspire innovative approaches in exploiting the rich and unique African heritage as far as nature-derived drug discovery is concerned.

#### 3.1.2 Bridging the Education–Industry Gap

Once the education system has been strengthened for quality and competitiveness, African universities should prioritize collaborating with industry partners to address the continent’s health and economic disparities. Establishing strong ties with pharmaceutical companies and research centers will not only provide practical exposure for students but also ensure that academic training remains relevant to the evolving needs of drug discovery for the continent. Internships, joint research projects, and mentorship opportunities from industry professionals can serve as platforms for students to gain insights into industry workflows and establish professional networks that are critical to their future career advancements. To enhance industry uptake of the proposed partnership with African educational institutions, it is essential to present a clear and mutually beneficial value proposition. One key advantage for the pharmaceutical industry is access to a skilled talent pool from Africa, which can support the launch of new programs on the continent. This access helps mitigate delays caused by workforce shortages and reduces the costs associated with recruiting specialized expertise from outside Africa.

#### 3.1.3 Early Introduction of Medicinal Chemistry

Creating a cycle of perpetuity in the drug discovery sciences is critical and would need refocusing at how and at what stage learners are offered the opportunity to consider this field as a career. To cultivate a robust pipeline of medicinal chemists, it is imperative to introduce drug discovery concepts early in academic programs. Incorporating relevant subjects in late undergraduate or early postgraduate studies can demystify medicinal chemistry, foster foundational skills, instill sustained interest, and embed a culture of innovation and research from an early age. Drawing from successful international models, exemplified by European Union research and funding organizations, such as the Horizon-2020 and European Molecular Biology Laboratory, African education systems should also integrate science outreach initiatives at primary and secondary levels to identify talent and inspire curiosity.^27^ Citizen science projects, school science fairs, and interactive workshops are crucial community engagement initiatives that can play a pivotal role in promoting medicinal chemistry as a vital contributor to societal development. Outreach programs tailored for schools and the public can raise awareness about the impact of science, technology, and innovation on socioeconomic and health outcomes, thereby showcasing it as a powerful resource to address societal challenges.^28^ Such programs have been integrated into various international funding mechanisms, including those supported by the European Union, as part of community and social engagement efforts to make science more accessible. Allocating budgets within these funding streams for African grant recipients would facilitate the implementation of similar initiatives across the continent. Additionally, tailored financing options, such as support from local governments and collaborations with science outreach and communication organizations, can further enhance the reach and impact of these programs. By highlighting success stories and career opportunities in drug discovery, these programs can encourage greater enrollment in drug discovery-related disciplines, ultimately contributing to the development of a skilled workforce capable of addressing Africa’s unique health challenges.

### 3.2 Funding, Government Policies, and Strategic Resource Utilization

Designing and execution of medicinal chemistry projects in Africa present significant challenges, particularly in establishing the necessary infrastructure and securing chemicals, reagents, and specialized human resources. These hurdles are compounded by bureaucratic delays, such as slow procurement processes, taxes, and customs clearance for research materials.^29^ African governments must recognize the urgency of supporting medicinal chemistry research by waiving import duties, expediting customs clearance for research materials, and providing logistical incentives, such as government-backed distribution networks. Additionally, proactive government engagement with chemical and reagent suppliers would be instrumental in codeveloping sustainable solutions with key stakeholders.

A strategic approach to building capacity in medicinal chemistry is the establishment of select centers of excellence across the continent. Modeled after initiatives like the Pan African University^30^ and World Bank African centers of excellence,^31^ such hubs could provide an integrated environment for advanced research and training. By consolidating resources, funding, and expertise within these hubs, Africa can cultivate a steady pipeline of skilled professionals while addressing systemic barriers through coordinated action. Furthermore, expanding the ecosystem of medicinal chemists within the proposed centers of excellence would create economies of scale, strengthening the business case for attracting chemical and reagent suppliers to strategic regions. This, in turn, would help mitigate challenges related to long lead times in the delivery of laboratory consumables, chemicals, and reagents.

Collaborative funding models offer another pathway to amplifying research impact. Pooling resources from smaller grants through consortia or partnerships can enhance support for early career researchers and optimize the use of limited funding. Such frameworks not only facilitate resource sharing but also help prevent duplication of efforts, enabling more efficient and impactful outcomes. Leveraging these models across centers of excellence will ensure sustainable and high-quality output in drug discovery.

International funding bodies play a central role in supporting African researchers. However, these opportunities often fail to account for the unique challenges faced by researchers on the continent. For instance, the requirement, by some prestigious funding instruments, for a well-qualified home supervisor often disadvantages candidates from disciplines such as medicinal chemistry, where such expertise is scarce. It is essential to establish funding streams that accommodate these realities, ensuring equitable access for early career African researchers, particularly in underrepresented fields. In such cases, having a suitable host supervisor in the proposed training institution should be considered adequate, and the need for an equivalent home supervisor, where this is lacking, should be waived, acknowledging that the applicant may be a trailblazer in their field and have no suitable mentor in their home organization.

Governments must also commit to framing medicinal chemistry as an economic and social asset. By incentivizing investments in biotech startups, forging partnerships with international organizations, and supporting the establishment of spin-off companies, African countries can position drug discovery as a viable economic sector. Viewing medicinal chemistry as a profession with the potential to generate income while addressing critical health needs will unlock further investment and stimulate innovation.

### 3.3 Research Collaborations and Mentorship

#### 3.3.1 Collaborations

With the current sparse distribution of medicinal chemistry-driven drug discovery research across Africa, collaborations are critical in enhancing this capacity.^32^ Several laboratories across the continent can conduct biological screening for potency determination, but the low level of synthetic organic chemistry capacity leaves such potential underutilized. Returning postgraduate trainees from overseas often struggle to adapt productively in their home institutions. Many give up on practicing their specialized skills and either concentrate on teaching and administrative duties or resort to conforming to other less specialized or less infrastructure-intensive research permissible in their environment. Usually, the lack of analytical equipment such as nuclear magnetic resonance (NMR) spectrometers and high-performance liquid chromatography–mass spectrometers (HPLC-MS) poses considerable obstacles to many medicinal chemists from venturing into early drug discovery hit-to-lead projects. Deliberate efforts to mount South–South and North–South collaborations can provide a feasible option to circumnavigate the challenges associated with access to specialized and expensive platforms. Establishing links with research centers within and outside the continent, and seeking research grants and funding, as part of these partnerships, can allow for reciprocal collaborative ventures enabling structure elucidation, characterization, and biological testing of synthesized compounds.

The proposed research collaborations can take the form of student research visits to laboratories where a unique drug discovery service, lacking in the home institution, is available. Such student visits, besides being crucial in overcoming limitations associated with shipping of samples, such as protracted lead times, accords a rare chance to scientists from resource-poor institutions to gain hands-on experience on the research, administrative, and science communication behaviors of the established research centers that play host to them. Implementing positive learning experiences upon return to the home organization can expedite the progress in research activities and further spearhead quality enhancement in the science to globally competitive standards.

Significant efforts have been made by some African research centers to procure and install specialized equipment for drug discovery projects, often through major grants or donations. However, sustaining the functionality of these pieces of equipment remains a major challenge, particularly in terms of maintenance and repair. In many cases, technicians must be flown in from overseas or South Africa, with costs that are often beyond the reach of institutions. As a result, once-promising equipment is left idle and unused, while the reliance on imported expertise leads to prolonged downtime.

Addressing this challenge requires holistic planning and a proactive investment in local capacity building. Training biomedical and instrumentation technicians alongside scientific researchers should be prioritized to ensure routine maintenance and knowledge transfer. Over time, Africa must develop a skilled workforce capable of servicing and maintaining the critical equipment used in drug discovery, reducing dependency on external expertise, and ensuring the sustainability of research infrastructure.

#### 3.3.2 Mentorship Programmes

Learnings from more experienced researchers can help younger researchers avoid pitfalls and follow paths that expedite their career progression and the advancement of science on the continent. This mentorship can guide the design of feasible drug discovery projects by, for example, prioritizing more synthetically accessible compounds in a resource-constraint environment. Considering the complex and evolving nature of drug discovery and development research, mentorship of African scientists by scientists from institutions with a track record is essential to ensure success. Commendable efforts by The Global Health Mentorship Programme (GHMP), administered by H3D Foundation, need to be amplified across the continent.^33^ Aimed at accelerating the development of drug discovery research capacity and scientific leadership in Africa, the program has, so far, enrolled over 50 mentees who are matched with industry experts to provide mentorship across thematic areas ranging from the technical aspects of drug discovery and development to scientific communication and leadership. Moreover, while such international mentorship schemes are key in building an international network of drug discovery researchers, more African institutions need to adopt in-house mentorship programs that respond to the need for education and professional guidance for early career scientists. The aspiration of such an ambitious mentorship arrangement is complicated by the lack of senior drug discovery scientists in many African countries. Where available, the senior drug discovery scientists may be overwhelmed by teaching and administrative workloads with little or no time spared for mentorship.

### 3.4 Establishing an African Medicinal Chemistry Knowledge and Capability Repository

The potential for greater drug discovery research output in Africa is hindered by a poor collaboration culture, both at the institutional level and within departments of the same institution. In addition to sensitizing African scientists on the need for strengthening collaborations, creating a repository of drug discovery capabilities and making such a repository public is pivotal. Utilizing such a repository can speed up collaboration endeavors, leading to faster progress of projects and reduce individual costs that would have been borne by researchers working in silos. The GC ADDA consortium is one such effort to promote collaborations across Africa.^26^

Development of databases for natural and synthetic compounds in Africa can be resourceful in promoting continental drug discovery efforts. While there have been efforts to establish virtual libraries of natural product compounds isolated from organisms in Africa,^34–39^ there is need to establish a library of actual physical samples of natural products and synthetic compounds. Apart from providing useful starting points for virtual screening, such a repository would also be resourceful in providing tool compounds for *in vitro* high throughput screening to identify hits for diverse drug discovery projects.

### 3.5 Afrocentric Conferences and Workshops

Organizing focused conferences and workshops that recognize the unique challenges experienced by scientists operating in Africa is a viable approach toward helping them navigate the drug discovery terrain. In addition to providing a learning environment, especially for early career researchers, such meetings also serve as platforms to encourage networking. These initiatives, exemplified by H3D symposiums^16^ have offered many first-time drug discovery researchers insights into topics such as screening of compound libraries, ADME (absorption, distribution, metabolism, and excretion) assays, mode of action studies, medicinal chemistry optimization, natural products drug discovery, and the use of artificial intelligence (AI) and machine learning (ML) in drug discovery. This exposure and knowledge may create further career development and initiation of research networks.

### 3.6 Leveraging Emerging Technologies

#### 3.6.1 Computer-Aided Drug Discovery

Drug discovery is an expensive undertaking, a fact which can be demoralizing to early career researchers hoping to venture into this field.^40^ In addition to engaging in strategic collaborations and partnerships, early career researchers can circumvent the costs associated with the early phases of drug discovery by employing computer-aided drug design (CADD) approaches. As an example, high-throughput virtual screening of large virtual compound libraries can aid in identifying hit compounds that can be subsequently progressed through to *in vitro* hit confirmation and further target-based medicinal chemistry optimization.

#### 3.6.2 Artificial Intelligence

Recent times have experienced an explosion in the use of AI in reducing costs and accelerating the drug discovery and development process.^41–44^ For instance, AI has been deployed in predicting three-dimensional structures of target proteins; designing multitarget drug molecules, designing synthetic routes, predicting new therapeutic use of existing drugs, and predicting toxicities and physicochemical parameters among other applications.^45^ It is evident that modern drug discovery approaches will accelerate utilizing advancements in technology, a resource that early career researchers in resource-poor settings ought to invest in, as this could considerably minimize costs and expedite progression of drug discovery projects. The collaboration between Ersilia Open Source Initiative (a technology nonprofit focusing on the dissemination of data science tools in resource-poor settings)^46^ and H3D, which introduced AI/ML tools in the latter’s research programs, is an excellent example illustrating the application of AI in accelerating drug discovery programs in resource-constrained settings. This AI/ML approach resulted in the development of the ZairaChem pipeline,^47^ which contains a suite of AI models with the capacity to predict compounds with drug-like properties. As suggested for other drug discovery capabilities, selected centers of excellence can be assigned the responsibility for strengthening and consolidating their expertise in CADD and AI/ML and offering this service to other consortium members to add value to the overall drug discovery projects from Africa.

#### 3.6.3 Drug Repurposing and Repositioning

In poorly resourced research environments, adopting strategies that circumvent the huge cost associated with *de novo* drug discovery and development is important. Finding new therapeutic use for an originally approved drug without making chemical modifications (drug repurposing) or after structural optimization (drug repositioning) is a powerful approach with potential cost and time savings. The use of drug candidates that have been through several stages of clinical development, with well documented pharmacokinetic and safety profiles, has tremendous potential to derisk the drug discovery and development process.^48^ Moreover, use of AI/ML models can further shorten the drug development time frame in drug repurposing efforts.^45,49^

## 4 Conclusions

In summary, Africa is on the cusp of a scientific renaissance, with burgeoning potential in medicinal chemistry and drug discovery to address its unique public health challenges. Exemplary centers, such as H3D at UCT, offer a blueprint for success, demonstrating how locally driven innovation can yield a global impact. However, scaling such efforts requires robust collaboration among African scientists, leveraging global networks and resources to build capacity and overcome persistent challenges.

Central to this transformation is the reimagining of education systems to integrate medicinal chemistry disciplines early in curricula, emphasizing practical, real-world applications that address the continent’s healthcare needs. Coupled with strategic investments in African-sensitive funding mechanisms, citizen science initiatives, and infrastructure development, these measures can catalyze a sustainable ecosystem for medicinal chemistry on the continent.

Africa’s vast biodiversity and rich natural resources, combined with advancements in computational tools such as AI and ML, provide unprecedented opportunities to fast-track drug discovery efforts. Establishing collaborative platforms such as an African Medicinal Chemistry Knowledge Repository or pan-African consortia could further enhance resource sharing, mentorship, and knowledge exchange.

Going forward, it is imperative that policymakers, academic institutions, and international partners unite to prioritize African-led initiatives, fostering a future where the continent not only meets its healthcare needs but also contributes to global drug discovery efforts. Doing so will ensure that Africa steps fully into its role as an equal leader in medicinal chemistry and innovation.

## Abbreviations Used

ADME: absorption, distribution, metabolism and excretion
AI: artificial intelligence
AiBST: African Institute of Biomedical Science and Technology
AREF: Africa Research Excellence Fund
BVGH: BIO Ventures for Global Health
CADD: computer-aided drug design
CDIPD: Centre for Discovery and Innovation in Parasitic Diseases
DMPK: drug metabolism and pharmacokinetics
GC ADDA: Grand Challenges African Drug Discovery Accelerator
GHMP: Global Health Mentorship Program
H3D: Holistic Drug Discovery and Development Center
HPLC-MS: high-performance liquid chromatography – mass spectrometer
JKUAT: Jomo Kenyatta University of Agriculture and Technology
ML: machine learning
NGS: Next Generation Scientist
R&D: research and development
NMR: nuclear magnetic resonance
UCL: University College London
UCSD: University of California San Diego
UCT: University of Cape Town
UNZA: University of Zambia
WACCBIP: West African Centre for Cell Biology of Infectious Pathogens
WIPO: World Intellectual Property Organization
